# SRRM4 Knockout Helps the Human Mesenchymal Stem Cell Line to Penetrate Decellularized Cancellous Bone

**DOI:** 10.3390/bioengineering12121299

**Published:** 2025-11-26

**Authors:** Iichiroh Onishi, Karin Muraoka, Anri Koyanagi, Tsuyoshi Kimura, Akio Kishida, Masanobu Kitagawa, Morito Kurata

**Affiliations:** 1 Department of Pathology, Institute of Science Tokyo Hospital, 1-5-45 Yushima, Bunkyoku, Tokyo 113-8510, Japankuratapath@gunma-u.ac.jp (M.K.); 2 Department of Comprehensive Pathology, Graduate School of Medical and Dental Sciences, Institute of Science Tokyo, Tokyo 113-8519, Japan; 3 Department of Biomedical Engineering, Faculty of Life Sciences, Toyo University, Saitama 351-8510, Japan; 4 Laboratory for Biomaterials and Bioengineering, Institute of Integrated Research, Institute of Science Tokyo, Tokyo 101-0062, Japan

**Keywords:** bone marrow, decellularized bone (DCB), bone marrow mesenchymal stem cell (MSC) line, CRISPR knock out library, Serine/Arginine Repetitive Matrix 4 (SRRM4)

## Abstract

Background: Analyzing the human bone marrow microenvironment requires an in vivo model that reflects the human bone marrow microenvironment. Introducing a human bone marrow mesenchymal stem cell (MSC) line into decellularized cancellous bone (DCB) is a first step in forming such a bone marrow model. Our goal with this research is identifying factors that promote the penetration of MSCs into DCBs in an ex vivo setting. Methods: We introduced the CRISPR Knock Out (GeCKO v2) library to identify candidate genes in UE7T-9 cell line (MSC line) for DCB penetration. We established a candidate gene-knockout UE7T-9 cell for validation and evaluated its penetration into DCB (measured distance of randomly selected 100 cells), proliferation (MTS assay), migration (scratch assay), and ancorage-independent growth (soft agar assay). RNA sequencing was performed to analyze changes in gene expression comprehensively. Results: We identified *Serine/Arginine Repetitive Matrix 4 (SRRM4)* knockout (KO) in the UE7T-9 cell as a candidate factor for bone penetration. *SRRM4* KO promoted DCB penetration (3.1–7.1 times deeper, each *p* ≤ 1.91 × 10^−24^), cell migration (*p* = 0.039), and ancorage-independent growth (2.5 times in colony count, 7.1 times in colony size, each *p* = 0.001) but retained stem cell characteristics. Conclusions: *SRRM4* KO is a newly defined factor of UE7T-9 cell penetrating into DCB. *SRRM4* KO UE7T-9 cells may be used to analyze hematological diseases such as myelodysplastic neoplasms.

## 1. Introduction

Human hematopoiesis occurs within the bone marrow, where the differentiation of erythrocytes, leukocytes, and platelets is regulated and produced in large numbers. The hematopoietic stem cells (HSCs) that give rise to these cells maintain their differentiation and proliferation in the bone marrow microenvironment through contact and interaction with bone marrow mesenchymal stem cells (MSCs), adipocytes, osteoblasts, and blood vessels. Abnormalities in the contacts and interactions between HSCs and these mesenchymal cells contribute to hematopoietic abnormalities and tumorigenesis of HSCs [1]. In some cases of acute leukemia and myelodysplastic neoplasms, the interaction between HSCs and bone marrow-derived MSCs with genetic abnormalities within the bone marrow microenvironment may induce HSC tumorigenesis [2]. Experiments using mice have reported an association between genetic mutations, such as *Dicer1* in MSCs, but not in HSCs, resulting in myelodysplastic neoplasms [3]. Myelodysplastic neoplasms have a wide range of pathogenesis and often progress to acute myelogenous leukemia. It often occurs in older individuals, and the number of patients is expected to increase with the aging population.

Therefore, a validated in vivo model reflecting the human bone marrow microenvironment is needed for a detailed analysis of the bone marrow microenvironment, in which human bone marrow mesenchymal cells can be introduced into experimental animals [4].

One method involves creating bone-like scaffolds from chemicals or living cells, introducing human hematopoietic cells, and transplanting them into experimental animals.

Artificial bone substitutes, such as tricalcium phosphate-hydroxyapatite, biphasic calcium phosphate, and polycaprolactone, have been used to induce osteoblasts [5], and human bone marrow mesenchymal cells have been transplanted into immunodeficient NSG mice to induce differentiation into bone and bone marrow tissue [6]. The former is problematic because of its low efficiency of introduction into artifacts, whereas the latter is problematic because of its random degree of bone formation. Additionally, both require many primary cultured cells derived from patients or healthy donors, are difficult to label with green fluorescent protein, and introduce genetic mutations such as clustered regulatory interspaced short palindromic repeats (CRISPR). These problems make it difficult to reconstruct the pathological bone marrow microenvironment with genetic mutations in bone marrow mesenchymal cells and to distinguish between human- and mouse-derived cells mixed in decellularized bone after transplantation.

Scaffolds made of natural collagen fiber (collagen) [7] and hydroxyapatite [8] induce MSCs differentiation to osteoblasts. These natural scaffolds are mainly used for one component and limiting differentiation of MSC.

In contrast, the method using decellularized cancellous bone (DCB) involves the application of high hydrostatic pressure to porcine ribs to remove cellular components. DCB retains trabecular structure, allowing porcine bones to be shaped as desired while maintaining strength. It also preserves adequate space for cell induction. After DCB transplantation into the rat subcutaneous tissue, the hematopoietic system was induced within the DCB. Mesenchymal cells include osteoblasts, adipocytes, hematopoietic cells, and HSCs [9]. Because DCB had signal molecules remaining on the decellularized extracellular matrix, rat mesenchymal stem cells and HSCs were induced and differentiated. This technique enables the subcutaneous implantation of biomaterials from different animal species in mice and rats, thereby inducing an autonomous bone marrow environment without eliciting a foreign body reaction in the host.

By implanting human MSC lines into the DCB and transplanting them into mice, it is possible to induce an artificial bone marrow microenvironment in the DCB consisting of hybrid, human-oriented MSCs, mesenchymal cells, and mouse-induced hematopoietic cells without eliciting foreign body reaction. Human MSCs autonomously differentiate into other mesenchymal cells, such as adipocytes, vascular cells, and osteoblasts, under bioactive conditions in mouse subcutaneous tissue [10]. Therefore, human bone marrow MSC lines with gene mutations, such as *DICER1*, introduced into the DCB before transplantation and subcutaneously transplanted into mice, could be used to create a new model of human leukemia-prone bone marrow. For this aim, using DCB and mesenchymal stem cell line is a solution with advantages over the problems of other artificial bone substitutes.

To create a human bone marrow microenvironment, MSC lines (e.g., UE7T-9 cells derived from the bone marrow) must be engrafted into the DCB. Culturing DCB and cell lines alone do not allow their efficient penetration into DCB. In contrast, Nakamura et al. reported that low-temperature human immortalized MSCs seeded onto DCB were effective because of reduced cell adherence ability [11]; however, continuing low-temperature conditions inhibited MSCs’ penetration and cell viability. We did not obtain sufficient engraftment of UE7T-9 cells. It is necessary first to identify the factors that introduce the bone marrow MSC line into DCB. The CRISPR library can be used to design guide RNAs (gRNAs) for approximately 18,000 genes (exon regions) [12] which can be used for the specific and convenient transfection of MSCs into decellularized bone. A CRISPR knockout (KO) library was used to comprehensively knock out each whole-exon gene by modifying the enzymes. We confirmed the viability of human MSCs in tissue samples and identified the responsible gRNAs.

Therefore, this study aimed to experimentally introduce human bone marrow mesenchymal cell lines into DCB using CRISPR KO library and evaluate the function of cells transfected with the identified genetic mutations in an ex vivo setting.

## 2. Materials and Methods

### 2.1. Cell Lines and Cell Culture

A human MSC line (UE7T-9 cells) was purchased from the Japanese Cancer Research Bank (JCRB, Osaka, Japan) cell bank in 2014 (https://cellbank.nibiohn.go.jp/~cellbank/cgi-bin/search_res_det.cgi?ID=3821, accessed on 30 June 2025). Human embryonic kidney cells (HEK 293T) were purchased from the JCRB. UE7T-9 and HEK 293T cells were maintained with Dulbecco’s modified Eagle’s medium (D-MEM, Fujifilm, Tokyo, Japan) supplemented with 10% fetal bovine serum (Thermo Fisher Scientific, Waltham, MA, USA) and 1% penicillin/streptomycin (Thermo Fisher Scientific), incubated at 37 °C with 5% CO_2_. DMEM was used as the culture medium for all the experiments. Cell culture and all assays were performed in static condition.

### 2.2. Transfection of GeCKO v2 Library into Cell Lines

HEK 293T cells (5 × 10^5^ cells/well) were seeded in a six-well cell culture plate (Thermo Fisher Scientific) a day before performing the lentiviral transfection. The next day, 3 µg of lentivirus plasmid was transfected with 1 µg of pMD2.G and 2 µg of pCMV using Lipofectamine™ 3000 Reagent (Thermo Fisher Scientific), according to previous work [13]. Twelve hours after transfection, the medium was changed to fresh D-MEM. The virus supernatant was harvested 48 h after transfection and then filtered with Millex™-HP 0.45 µm (Merck, Kenilworth, NJ, USA). The target UE7T-9 cells (2 × 10^5^ cells/well) were seeded in a six-well cell culture plate one day before transduction. The cells were transduced with this lentivirus supernatant with 5 µg/mL polybrene (Sigma-Aldrich, St. Louis, MO, USA). Transduction was performed using spin infection and centrifugation at 4680 rpm for 30 min. The lentiviral plasmids used were Genome-scale CRISPR Knock Out, GeCKO v2.0 pooled Library (Addgene #1000000048, Watertown, MA, USA), and lentiCas9-Blast (Addgene #52962). First, lentiCas9-Blast was transduced into UE7T-9 cells, and the transduced cells were selected using blasticidin (10 μg/mL, Thermo Fisher Scientific, Waltham, MA, USA) for 2 weeks. hCas9-expressing UE7T-9 cells were used for further experiments. The CRISPR-activated library, lenti-GeCKO v2.0, was also transduced into hCas9-expressing UE7T-9 cells. Although the multiplicity of infection is usually set to one or less, in this study, we allowed duplicate infections (multiplicity of infection > 1) because our goal was to invade the DCB. We then performed selections with puromycin (1 μg/mL, InvivoGen, San Diego, CA, USA) for 2 weeks.

### 2.3. DCB Formation

Bones were prepared as previously described [9]. Fresh pig ribs were obtained from a local slaughterhouse (Tokyo Shibaura Shipbuilding). Ribs (including compact bones and trabecular bones) were cut into small pieces (15 mm × 15 mm × 4 mm. and washed in phosphate-buffered saline (PBS) (Invitrogen, Tokyo, Japan) containing penicillin (100 units/mL) and streptomycin (100 μg/mL). The bone marrow was filled with phosphate-buffered saline (PBS) and sealed in a plastic pack to prevent implosion and leakage during the application of pressure. The cells were dissociated by hydrostatic pressurization at 980 MPa and 30 °C for 10 min using a cold isostatic pressurizer (Dr. CHEF; Kobe Steel Ltd., Hyogo, Japan). Pressurization and decompression were performed at 65.3 MPa/min, and propylene glycol was used as the permeate. The cells were then washed with saline containing DNase I (0.2 mg/mL) (Roche Diagnostics, Tokyo, Japan) and an antibiotic for 3 weeks at 37 °C with continuous slow shaking and then treated with 80% *v*/*v* EtOH at 37 °C for 7 days. After washing and shaking with PBS again, they were stored at 4 °C.

### 2.4. Cell Culture on DCB and Histological Evaluation

To evaluate the penetration into DCB, UE7T-9 cells (2.0 × 10^5^ cells/well) were cultured on DCB in six-well cell culture plate for 3 weeks. After culturing, the cells were fixed in 10% neutral-buffered formalin for 1 d and demineralized in 18.5% EDTA solution (Pharma, Tokyo, Japan) for 1 d. Paraffin-embedded sections were prepared, and hematoxylin-eosin staining of thin sections (3 μm) was performed. The stained sections were photographed using a NanoZoomer S210 virtual slide scanner (Hamamatsu Photonics, Shizuoka, Japan).

### 2.5. DNA Sequence Analysis

Paraffin-embedded sections were thinly sliced at 10 μm. For five sections of DCB with surface cells removed, we added 0.5 mL of DEXPAT (TaKaRa, Shiga, Japan), heated at 100 °C for 10 min, and centrifuged at 4 °C at 12,000 rpm for 10 min. The aqueous layer was collected, avoiding the paraffin thin film, and purified using the Wizard SV Gel and PCR Clean-up System (Promega, Madison, WI, USA). For gRNA detection, PCR using the KOD-FX (TOYOBO, Osaka, Japan) method was then performed using GeCKOv2 82F: 5′-GAGGGCCTATTTCCCATGAT-3′ and GeCKOv2 278R: 5′-CGGTGCCACTTTTTCAAGTT-3′ as primers. The PCR products were purified and introduced into the Zero Blunt TOPO vector using the Zero Blunt TOPO PCR Cloning Kit (Thermo Fisher Scientific, Waltham, MA, USA). We performed DNA sequence analysis (Sanger method), identified gRNA, and searched for the corresponding genes in the gRNA list of the CRISPR GeCKO v2 Library.

(https://media.addgene.org/cms/filer_public/a4/b8/a4b8d181-c489-4dd7-823a-fe267fd7b277/human_geckov2_library_a_09mar2015.csv, accessed on 30 June 2025).

### 2.6. Creation of Serine/Arginine Repetitive Matrix 4 (SRRM4)-Knockout Cell Lines

The oligomers for the coding gRNA region of SRRM4 (SRRM4-5: CACCGATTACCTTGGCAGCGGCTTG, SRRM4-3: AAACCAAGCCGCTGCCAAGGTAATC) were purchased (Thermo Fisher Scientific, Waltham, MA, USA). Oligomers (100 µM) and T4 Polynucleotide Kinase (TaKaRa) were prepared and annealed. The annealed oligo was diluted 200-fold, ligated, and introduced into a lenti-CRISPR v2 (Addgene) plasmid following the manufacturer’s protocol (previous report [14]). After introducing this vector into *Escherichia coli*, subjected to selection with 200 μg/mL of ampicillin, the plasmid was recovered using a mini-prep, and the presence of the target band was confirmed by electrophoresis. Next, using this vector, Lipofection was performed as in (1) above, and the virus supernatant was infected with UE7T-9 (2.0 × 10^5^ cells/well) and subjected to selection with 1 μg/mL of puromycin.

After selection, UE7T-9 cells were adjusted to 1 cell/well in a 96-well plate, diluted, and cloned. The cell line obtained by cloning is hereafter referred to as the UE7T-9 SRRM4 KO cell line.

### 2.7. Flow Cytometry Analysis

The UE7T-9 cells and UE7T-9 SRRM4 KO (each 1.0 × 10^6^ cells) were fixed with 100 µL of 4% paraformaldehyde (Wako, Tokyo, Japan), permeabilized with 90% methanol (Sigma-Aldrich), washed twice with PBS, and incubated with the primary antibody for 1 h at room temperature. The samples were then incubated with the secondary antibody for 30 min at room temperature under light-shielded conditions and washed twice with PBS. Antibodies of SRRM4, CD73, CD105, and CD90 were listed in Table S1. Primary and secondary antibodies were each diluted 100-fold. A BD FACSCanto II Flow Cytometer (Becton Dickinson and Company, Franklin Lakes, NJ, USA) was used for detection.

### 2.8. Co-Culture with DCB and Measurement of the Penetration Distance

UE7T-9 SRRM4 KO (1.0 × 10^6^ cells) were co-cultured with decellularized bone in a 6 -well cell culture plate at 37 °C for four weeks under 5% CO_2_ control. After co-culture, paraffin-embedded sections were prepared, stained with hematoxylin and eosin, and photographed using a NanoZoomer S210 virtual slide scanner (Hamamatsu Photonics). The distance of penetration from the decellularized bone surface was measured using an NDP Viewer 2 (Hamamatsu Photonics). We measured penetration distance of 100 cells and performed three times using different lot of DCB. Statistical analysis was performed on each of the three groups.

### 2.9. Immunohistochemistry

A primary rabbit monoclonal antibody against anti-CXCL12 (clone D8G6H; Cell Signaling Technology, Danvers, MA, USA) was used in this study. FFPE tissue sections (4 mm thick) were deparaffinized in xylene and rehydrated through graded alcohol to water. Antigen retrieval pretreatments were 40 min microwave unmasking at 97–98 °C in staining buffer (pH 9.0, Nichirei Bioscience, Tokyo, Japan). Endogenous peroxidase activity was inhibited with 0.3% hydrogen peroxide in methanol for 30 min. Nonspecific protein binding was blocked by incubation with 10% normal horse serum (Vector Laboratories, Newark, CA, USA). The sections were then incubated with a primary CXCL12 antibody (dilution, 1:200) overnight at room temperature. After washing thrice with T-PBS, a secondary antibody (VECTASTAIN ABC Kit, Newark, CA, USA) was added. After being washed three times with T-PBS, detection was performed using the streptavidin–biotin–peroxidase complex technique (VECTASTAIN ABC Kit) and 3,3′-diaminobenzidine (DAB peroxidase substrate kit SK-4100, Vector Laboratories) as the chromogen. Counterstaining was performed using hematoxylin.

### 2.10. 3-(4,5-Dimethylthiazol-2-yl)-5-(3-carboxymethoxyphenyl)-2-(4-sulfophenyl)-2H-tetrazolium (MTS) Assay

An MTS assay was performed to evaluate cell proliferation. The UE7T-9 cells were cultured at a density of 5 × 10^3^ cells/well in a 96-well plate (Thermo Fisher Scientific). At 24, 48, and 72 h of culture, we added 20 µL of MTS (2 mg/mL) (Promega, Madison, WI, USA)/phenazine methosulfate (0.92 mg/mL) (Promega, Madison, WI, USA) to each well. The wells were then shielded from light and placed in an incubator for 2 h. Absorbance was measured at 490 and 650 nm using a microplate reader (ELx808; IU BioTek Instruments Inc., Thermo Fisher Scientific, Waltham, MA, USA). Data were statistically analyzed using Student’s *t*-test.

### 2.11. Scratch Assay

We used a scratch assay to evaluate cell migration ability. UE7T-9 cells were cultured at 3.0 × 10^4^ cells/70 µL using culture inserts (Ibidi, Gräfelfing, Germany). After 24 h, the culture inserts were removed, and cells were cultured for 18 h. Formalin fixation and hematoxylin and eosin staining were performed. The area between the inserts after cell migration (cell-free area) was measured using the ImageJ 1.53k software (Wayne Rasband and contributors, National Institutes of Health, Bethesda, MD, USA). Data were statistically analyzed using Student’s *t*-test.

### 2.12. Soft Agar Assay

We used a soft agar assay to evaluate ancorage-independent growth. A 3.2% stock agarose solution was prepared using seaplaque agarose (Lonza, Basel, Switzerland). The stock agarose solution was mixed with DMEM to prepare a base agarose solution (0.8%). Then, 1.5 µL was dispensed into 6-well cell culture plate and allowed to solidify at room temperature. The stock agarose solution was mixed with UE7T-9 SRRM4 KO (1.0 × 10^4^ cells), and 1 µL was added to the base agarose layer and layered. After coagulation at room temperature, Dulbecco’s modified Eagle’s medium (1 mL/well) was added to each well, maintained at 37 °C and 5% CO_2_, and the upper layer of D-MEM was replaced every 7 days. After 17 days, colonies were stained with 4% formaldehyde/0.005% crystal violet solution and observed under an inverted fluorescence phase contrast microscope (KEYENCE, Osaka, Japan). The number and area of colonies were determined using ImageJ 1.53 k.

### 2.13. Description of RNA Sequence Analysis

Total RNA was extracted from UE7T-9 SRRM4 KO and UE7T-9 cells using the RNeasy Mini Kit (QIAGEN, Hilden, Germany). All library preparations and sequencing were performed by Novogene Japan K.K. (Tokyo, Japan) using the NovaSeq 6000 Sequencing System (Illumina, San Diego, CA, USA). We performed mRNA-seq (Non-Stranded). For the volcano plot, we utilize the ggplot2 package in R. For Gene Set Enrichment Analysis (GSEA), normalized expression data were analyzed and visualized using the GSEA software (version 4.2.3, https://www.gsea-msigdb.org/gsea/index.jsp, accessed on 30 September 2025). The normalized enrichment score, nominal *p*-value (value estimating the statistical significance of enrichment score for a gene set), and false discovery rate q-values (an indicator estimating the proportion of false positives among significant results found when analyzing multiple gene sets) were calculated for comparison on GSEA software, and the selected categories were universally upregulated in UE7T-9 *SRRM4* KO cells. The relative enrichment of individual genes was assessed based on the rank metric score following GSEA.

### 2.14. Statistical Analysis

Data were statistically analyzed using GraphPad Prism Version 9.3.1 (350) software. All experiments were performed at least three times, and the results are expressed as the mean ± standard deviation. Statistical significance was determined using the Mann–Whitney U test in measurement of the penetration distance and Student’s *t*-test in MTS assay, scratch assay, and soft agar assay. Statistical significance was set at *p* < 0.05 for all analyses.

## 3. Results

### 3.1. Finding Candidate Factors for Penetration into DCB Using the CRISPR GeCKO v2 Library

**Figure 1:**
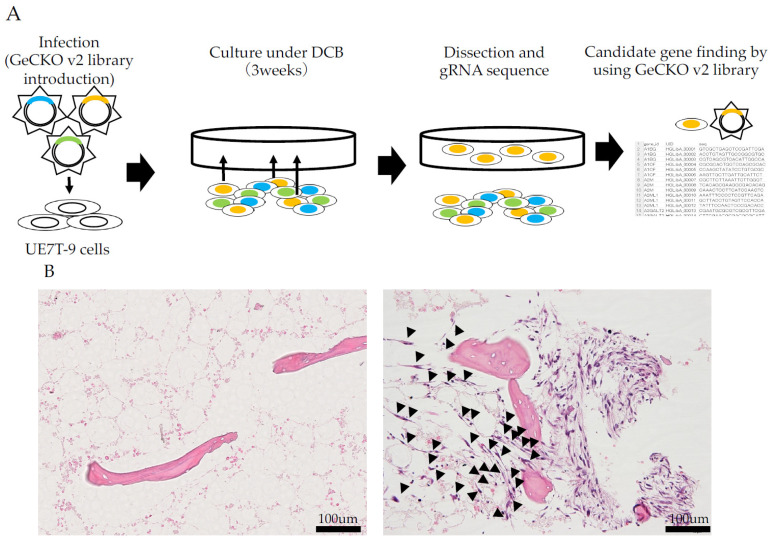
Introduction of CRISPR v2 library to UE7T-9 cells and co-culture under DCB. (**A**) Schematic illustration of the experiments using CRISPR KO library: CRISPR v2 library vectors were introduced in the host cell (HEK293T) and packaged in lentivirus vector. UE7T-9 cells were infected with Lentivirus vector containing each gRNA (blue, yellow, and green). After 3 weeks of culture of UE7T-9 cells under DCB, DCB was fixed in formalin and paraffin embedded. Thin-sliced sections were dissected to leave UE7T-9 cells penetrating into DCB. Remained UE7T-9 cells were DNA-extracted and sequenced in the gRNA region. (**B**) Histology of UE7T-9 cells were transfected with GeCKO library: UE7T-9 cells without GeCKO library showed no penetration into decellularized bone parenchyma (left), whereas UE7T-9 cells with GeCKO library showed a cohesive penetration into the bone marrow parenchyma (right: black arrowheads). The bar is 100 µm. The penetrating component of the parenchyma was dissected and used for subsequent gRNA analysis.

A schematic representation of this procedure is shown in Figure 1A. The GeCKO v2 library was introduced into UE7T-9 cells via infection with a lentiviral vector. After 3 weeks of co-culture under DCB with UE7T-9 cells transfected with the GeCKO v2 library, cells penetrated into the marrow of DCB histologically, whereas control cells did not (Figure 1B). The penetrating cells were spindle-shaped and showed no apparent differentiation.

We obtained penetrated cell samples from FFPE slides, omitted surface cells by microdissection, and extracted gDNA. The gRNA sequences used in this study are listed in Table 1. As listed in Table 1, three clones were *SRRM4* gRNA sequence and 4 clones were not found. Only *SRRM4* KO cells were identified among the UE7T-9 cells.

Next, we established UE7T-9 SRRM4 KO cells as described in Section 2. Although we attempted to validate the *SRRM4* KO using Western blotting, flow cytometry showed reduced SRRM4 expression (Supplementary Figure S1). However, we could not obtain complete KO cells. The experiment used these KO cells as the UE7T-9 SRRM4 KO cells.

### 3.2. Establishment of SRRM4 KO UE7T-9 Cells and Variation

**Figure 2:**
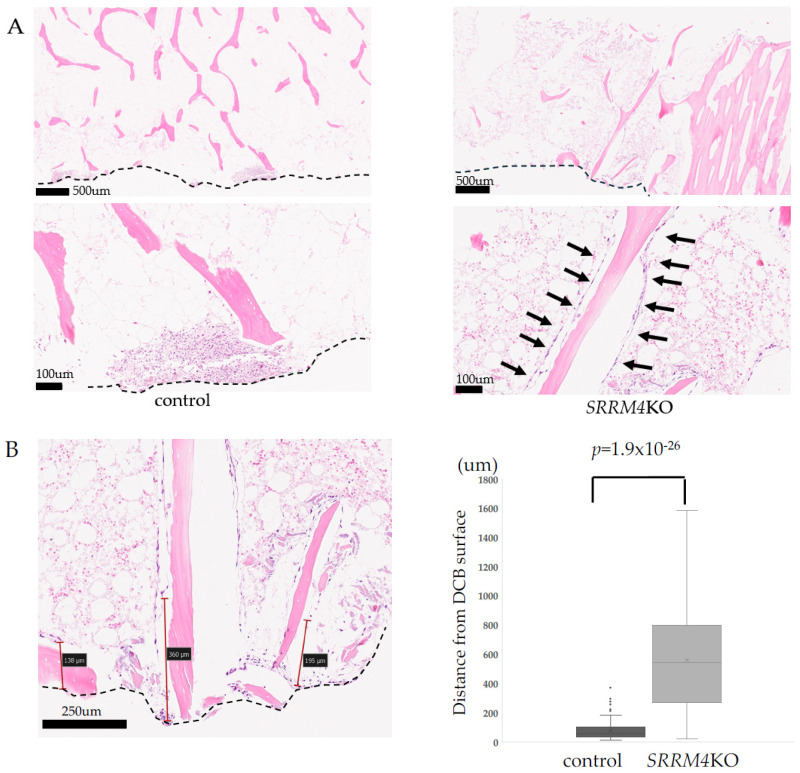

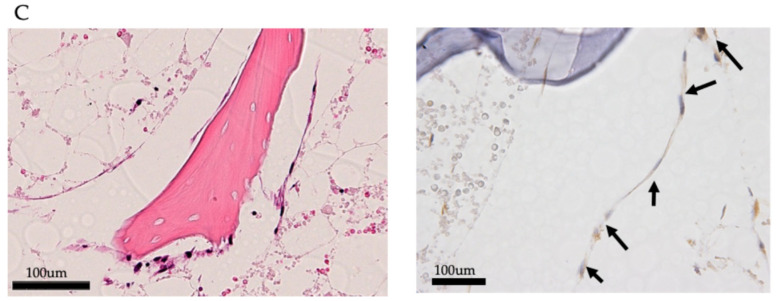
DCB intrusion of UE7T-9SRRM4 KO cells. (**A**) UE7T-9 cells extend into decellularized bone. Dotted line is surface of DCB. Upper and lower left: Control shows no extension into the bone trabeculae or bone marrow. Upper and lower right: UE7T-9 SRRM4 KO cells extend continuously along the bone trabeculae. Black arrows: Penetrating UE7T-9 SRRM4 KO cells, Dotted line: DCB Surface, Black bars = 500 µm: upper pictures, 100 µm: lower pictures. (**B**) Distance measurements of penetration in UE7T-9 cells. Left: Distance of cells that extended from the surface of each DCB was measured (NDP viewer). 100 cells were randomly selected. Dotted line is surface of DCB. Black bar = 250 µm Right: In UE7T-9 SRRM4 KO cells, the cells significantly extended into the bone marrow. The measurement of the penetration distance was performed three times using different DCBs from individual porcin ribs. The data from one representative measurement is shown in the figure. (**C**) CXCL12 immunostaining of SRRM4KO UE7T-9 cells extended into the bone matrix (left: HE stained. Right: CXCL12 immunohistochemistry) UE7T-9 SRRM4 KO cells penetrated into the bone ramus show CXCL12 positivity. Black arrows: CXCL12-positive UE7T-9 SRRM4 KO cells, Black bars: 100 µm.

UE7T-9 SRRM4 KO cells and decellularized bone were co-cultured for four weeks, and HE staining was performed. In the control group, cells were identified only on the bone surface. In contrast, in SRRM4 KO mice, cells were observed along the bone trabeculae (Figure 2A). We measured the distance from the surface to the penetrating cells, and the SRRM4 KO was found to be significantly more deeply penetrating than the control (Figure 2B). In UE7T-9 cells, SRRM4 KO significantly promoted the penetration into the DCB (1: average 3.1 times deeper, *p* = 1.9 × 10^−24^, 2: average 7.1 times deeper, *p* = 1.9 × 10^−26^, 3: average 5.1 times deeper, *p* = 1.9 × 10^−24^). Penetrating UE7T-9 SRRM4 KO cells retained CXCL12 expression (Figure 2C), suggesting that these cells retained MSC properties.

### 3.3. Evaluation of Migration, Proliferation, and Transformation

**Figure 3:**
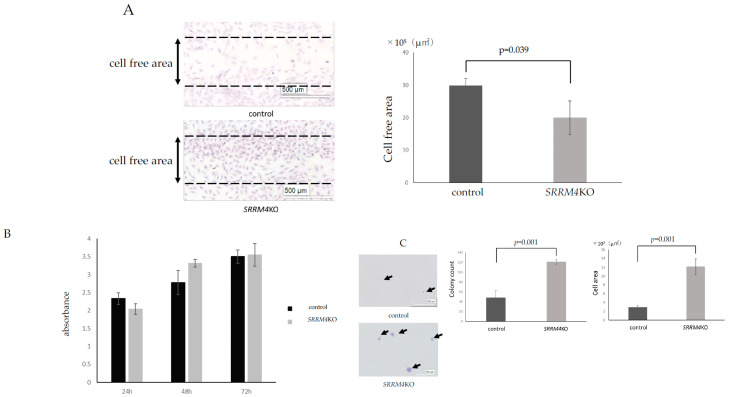
Cell migration, proliferation and anchorage-independent colony formation of UE7T-9 SRRM4 KO cells. (**A**) Scratch assay of UE7T-9 cells Left: UE7T-9 cell control and UE7T-9 SRRM4 KO cells were fixed and HE stained 15 h after removal of culture inserts. Assays were performed three times. White bars: 500 µm. (**B**) MTS assay of UE7T-9 cells Cell counts of UE7T-9 cell control and UE7T-9 SRRM4 KO cells were evaluated by MTS assay after 24, 28, and 72 h of culture. No significant difference was observed in cell number and cell proliferation in both cells. Assays were performed three times. (*p* = 0.086: 24 h, 0.059: 48 h, 0.84: 72 h). (**C**) Soft-agar assay of UE7T-9 cell. Left: Anchorage-independent proliferation of UE7T-9 cell control and UE7T-9 SRRM4 KO cell was evaluated by soft agar assay. (Black Arrows: cell cluster, White bars: 500 µm). Middle: Colony count of UE7T-9 cell Control and UE7T-9 SRRM4 KO cell. Right: Colony scale of UE7T-9 cell Control and UE7T-9 SRRM4 KO cell. Assays were performed three times.

Increased migratory ability is thought to favor bone penetration. Therefore, we evaluated the role of *SRRM4* KO in cell migration using a scratch assay. Culture inserts were removed, fixed for 15 h, and stained (Figure 3A). Compared with controls, the cell-free area was significantly reduced in SRRM4 KOs (*p* = 0.039).

The MTS assay was performed to evaluate whether bone invasion was influenced by differences in cell proliferative capacity. Figure 3B shows no significant differences between controls and *SRRM4* KO at 24, 48, or 72 h. The SRRM4 KO did not influence the proliferation of UE7T-9 cells.

For progression into bone, cells must detach from the scaffold of the culture dish and proliferate within the bone matrix, which is less stable than the culture dish. In order to address the anchorage-independent growth, we also performed a soft agar assay (Figure 3C). Compared to control cells, UE7T-9 SRRM4 KO cells showed a significantly increased colony count and colony size (2.5 times in colony count, 7.1 times in colony size, each *p* = 0.001). These results show that *SRRM4* KO promoted the migration capacity and anchorage-independent growth of UE7T-9 cells.

### 3.4. RNA Sequence Analysis of UE7T-9 SRRM4 KO Cells

**Figure 4:**
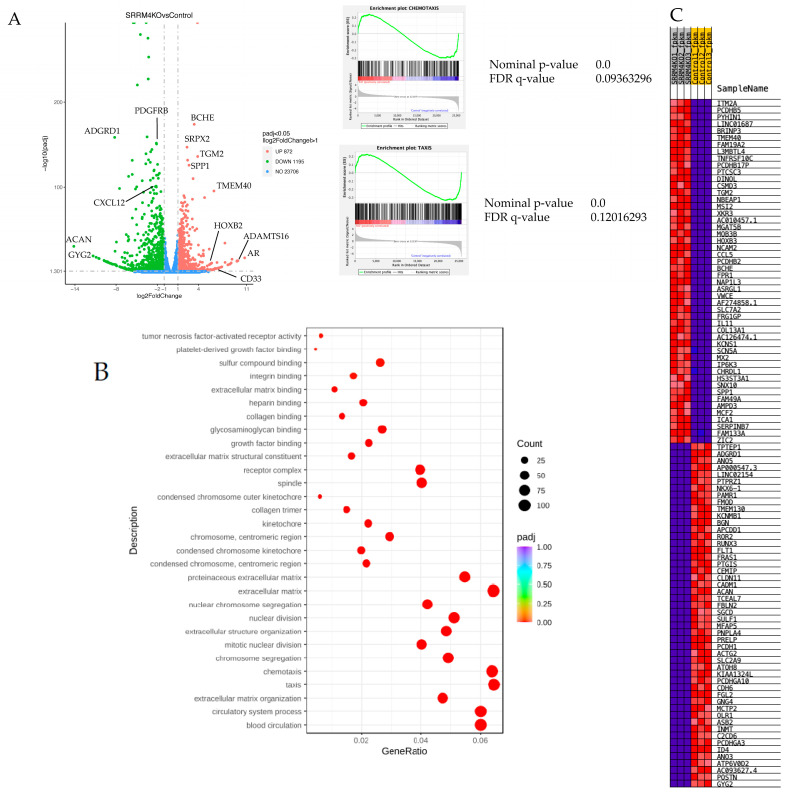
RNA sequence analysis of UE7T-9 and UE7T-9 SRRM4 UE7T-9 cells. (**A**) Left: Volcano plot identified upregulated and downregulated genes, including mild downregulation of CXCL12 and upregulation of HOXB2, but no obvious differentiation factors or growth factors were identified. However, no obvious differentiation factors or cell growth factors were identified. Right: GO Gene ontology analysis showed upregulation of factors such as chemotaxis and extracellular matrix. KO cell. (**B**) Pathway enrichment analysis shows the pathways to which the differential genes are most related, with reference to the Gene Ontology database. (**C**) Heat map of the upregulated genes and downregulated genes between UE7T-9 cell control and UE7T-9 SRRM4 KO cell.

We comprehensively analyzed the gene expression changes in UE7T-9 cells by SRRM4 KO using RNA sequencing (Figure 4A). A total of 872 genes were upregulated, and 1195 genes were downregulated. We found that SRRM4 KO increased the expression of the androgen receptor, myeloid cell marker CD33, and mildly decreased the expression of CXCL12. ACAN (aggrecan) and glycogenin 2, markers of osteochondrogenic differentiation, were downregulated. No other differentiation markers were found to be upregulated. GSE analysis showed enrichment of cell migration factors, such as chemotaxis, taxis, circulatory system processes, and blood circulation (Figure 4B,C, Supplementary Figure S2), supporting the increased migration ability in the scratch and soft agar assays.

Although GSEA revealed bone marrow mesenchymal cells, osteoblast differentiation, chondrocyte differentiation, myogenesis, and adipogenesis, a certain induction of differentiation by *SRRM4* KO was noted (Supplementary Figure S2). Immunostaining of UE7T-9 *SRRM4* KOs expanded into the bone showed that CXCL12 expression was preserved (Figure 2C), indicating that they retained bone marrow-derived MSC characteristics. We further confirmed using flow cytometry that SRRM4 KO did not affect the expression of CD73 and CD105 (Mesenchymal stem cell markers) and showed little expression of CD90 (Hematopoietic stem cell marker) (Figure S3). We tried to evaluate osteoblastic, chondroblastic, and adipogenic differentiation by immunohistochemistry, but we could not confirm each differentiation.

## 4. Discussion

The use of genetically engineered human MSC lines to reproduce various bone marrow microenvironments, the use of DCB that can be transplanted subcutaneously into mice, the ability to easily extract DCB, the ability to track chronological changes in the bone marrow microenvironment, and the ability to propose new treatment methods to normalize the bone marrow microenvironment are factors that make the bone marrow microenvironment model unprecedented. This paper describes the identification of factors that introduce MSC line into decellularized bone in an ex vivo setting. Using a CRISPR knockout library, this study identified SRRM4 KO as a novel penetration factor for UE7T-9 in decellularized bone.

SRRM4, which was identified using a CRISPR knockout library, is expressed in the central nervous system and is a splicing factor specific for neuronal differentiation [15]. There have been reports on the development of antisense nucleic acid drugs targeting SRRM4 for the treatment of small-cell lung cancer [16] and its association with autism spectrum disorder [17], and it has been suggested that silencing of SRRM4 in tumors may favor growth [18]. However, no association with bone marrow or hematopoiesis has been reported.

In this study, we compared protein expression in SRRM4 KO cell lines using Western blotting and flow cytometry, but could not confirm the complete loss of SRRM4, resulting in the analysis of KO cell lines with only half SRRM4 expression. This could be due to the low expression of SRRM4 in UE7T-9 cells, the possibility that both allele defects in SRRM4 were lethal, and the unstable increase in UE7T-9 expression after limiting dilution.

In contrast, flow cytometry showed reduced SRRM4 expression and penetrations along the bone trabeculae when co-cultured with decellularized bone. The penetration distance measurements showed significantly deeper results than those of the control. Thus, although a partial SRRM4 KO was performed, we believe it was sufficient for this study to penetrate into DCB.

Additionally, the cell-free area of the SRRM4 KO cell lines was significantly reduced in the scratch assay compared to that of the control cell lines. In contrast, no significant difference was observed in the cell proliferation experiment using the MTS assay. In the soft agar assay, there was a significant increase in the number and area of colonies, indicating that the depletion of SRRM4 expression enabled growth even under non-adherent conditions. These characteristics suggest that they favor intraosseous extension and are not simply due to cell proliferation.

RNA sequences were enriched in the TAXIS and CHEMOTAXIS gene sets, supporting the scratch and soft agar assay data. SRRM4 KO did not show distinct differentiation of UE7T-9 cells, suggesting that the UE7T-9 cells that penetrated into the DCB maintained their MSC characteristics. After subcutaneous transplantation into mice, these cells differentiate into osteoblasts, vascular endothelial cells, and adipocytes, constituting the bone marrow niche.

Compared to previous report [11] that human MSC penetration was limited to the surface of DCB, UE7T-9 SRRM4 KO penetrated more deeply along bone trabecular. We have previously identified the overexpression of the SHC4 gene using the CRISPR activation library [13]. Although SHC4 and SRRM4 are distinct functional genes, interestingly, SHC4 overexpression and SRRM4 Knock Out had features in common such as “cell migration” and “extracellular matrix” and did not affect cell proliferation. It suggests functional screening by CRISPR in UE7T-9 cells was highly effective. SRRM4 KO induced more cells along the bone trabecula than SHC4 overexpression, suggesting that SRRM4 KO is a more promising factor. Although the penetration distance may be insufficient, SRRM4 KO cells exhibit unique penetration along trabeculae, suggesting high affinity for the surface matrix of decellularized bone. We plan to grow the SRRM4 KO cell line produced in this study in decellularized bone and subcutaneously transplant it into mice to evaluate cell growth and action in humans and mice. The observation of penetration along trabeculae suggests that when transplanted into mice, MSCs may receive mouse-derived cytokines and cellular signals, leading to proliferation and differentiation. Furthermore, analyzing human MSCs near trabeculae is expected to facilitate the analysis of the hematopoietic microenvironment.

The lack of CD90 expression in UE7T-9 cell control, which fails to meet the International Society for Cellular Therapy’s definition criteria for MSCs (requiring expression of CD90, CD73, and CD105), is problematic. For the UE7T-9 control, possibilities include loss of CD90 during culture, potential alteration due to lentivirus infection, or issues related to the antibodies used in flow cytometry. Investigation of differentiation into adipocytes and osteoblasts is necessary (this was difficult to perform in this study). As future prospective, we plan to evaluate UE7T-9 as an MSC and also consider using cell lines like UE7T-13, where CD90 expression has been confirmed [19]. At least, the absence of marker expression differences between the SRRM4 KO and control suggests that SRRM4 KO does not promote differentiation.

The results of this study can be used to assess the efficacy of *SRRM4* KO in creating a full-scale human bone marrow niche model. If it is possible to induce specific genetic abnormalities such as *DICER1* in UE7T-9 [3], it is expected that a human–mouse hybrid bone marrow niche can be reproduced in mice, leading to the creation of a myelodysplastic neoplasm model and a more detailed analysis.

In this study, we successfully introduced a bone marrow mesenchymal stem cell line into DCB, which is difficult for cell introduction. But, this research has limitations: this study utilized a single cell line and a single biomaterial, and *SRRM4* KO was shown to be effective only under these limited conditions. Moving forward, numerous challenges remain, including determining whether SRRM4 KO is effective in human primary cells and patient-derived cells and comparing cell introduction in different biomaterials.

UE7T-9 SRRM4KO cells align in trabeculae, closely resembling the arrangement of osteoblasts. If differentiation into osteoblasts can be induced after cell introduction, promoting ossification on decellularized bone surfaces could lead to applications in bone repair and bone defect treatment. Regarding bone repair, research is advancing not only with 3D materials [7,8] but also with 4D-printed bone tissue scaffolds that change shape in response to near-infrared light [20]. Introducing bone marrow mesenchymal cell lines into these biomaterials and identifying the necessary factors for penetration using a CRISPR library are highly effective approaches.

## 5. Conclusions

By introducing the CRISPR KO library, we identified *SRRM4* KO as a novel penetration factor of UE7T-9 into decellularized bone, and *SRRM4* KO may favor penetration into decellularized bone by enhancing the migration ability and scaffold-independent growth. Further analysis of the SRRM4 KO cell line and subcutaneous transplantation of decellularized bone co-cultured with *SRRM4* KO cells is needed to validate the hypothesis that *SRRM4* KO cells can be used to construct bone marrow tissue.

## Figures and Tables

**Table 1:** Sequences of gRNAs obtained from UE7T-9 cells with the GeCKO library, penetrate into the bone marrow. Three clones were gRNA of SRRM4.

gRNA Sequence	Number of Clones	Candidate Gene
CCGCCGCTTTGCGCTCGGAG	4	Not found
ATTACCTTGGCAGCGGCTTG	3	*SRRM4*

## Data Availability

RNA sequence data for this study are available from the Gene Expression Omnibus (GEO) at [https://www.ncbi.nlm.nih.gov/geo/query/acc.cgi?acc=GSE302358 (accessed on 16 July 2025)]. Cord number is “GSE302358”. Raw fastq files and processed count matrices are available.

## Supplementary Materials

The following supporting information can be downloaded at https://www.mdpi.com/article/10.3390/bioengineering12121299/s1. Figure S1: Protein quantification analysis of SRRM4 KO cell line; Figure S2: Graphical views of the enrichment plot of the GSEA; Figure S3: Validation of differentiation of UE7T-9 SRRM4 KO cell; Table S1: Antibodies used for flow cytometry analysis; Table S2. Geneset members on the rank-ordered list of GO enrichment analysis of differential genes.

## Abbreviations

The following abbreviations are used in this manuscript:

MSC	Mesenchymal stem cell
DCB	Decellularized bone
HSC	Hematopoietic stem cell
SRRM4	Serine/Arginine repetitive matrix 4
