# Supplementary material for: SRRM4 Knockout Helps the Human Mesenchymal Stem Cell Line to Penetrate Decellularized Cancellous Bone
Source: Bioengineering (Basel). 2025 Nov 26;12(12):1299. doi: 10.3390/bioengineering12121299 (PMC12729399; doi:10.3390/bioengineering12121299)
Supplement: Supplementary file 1 [file bioengineering-12-01299-s001.zip › bioengineering-3839539-supplementary.pdf]

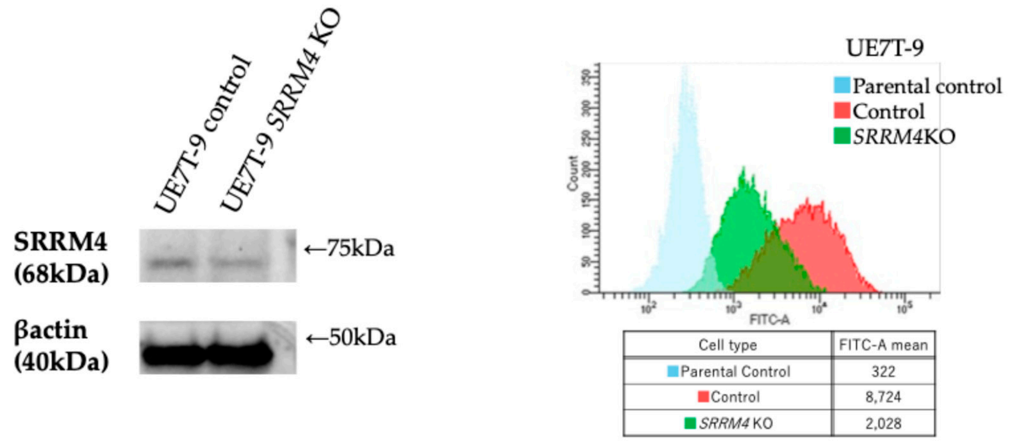

Cell type	FITC-A mean
Parental Control	322
Control	8,724
SRRM4 KO	2,028

Figure S1. Protein quantification analysis of SRRM4 KO cell line. Left :Western blot analysis showed that the SRRM4 KO cell line appeared to be down-regulated compared to the control, but there was no complete loss of protein expression. Right: Flow cytometry analysis showed reduced expression in the SRRM4KO strain (green) compared to the control (red).

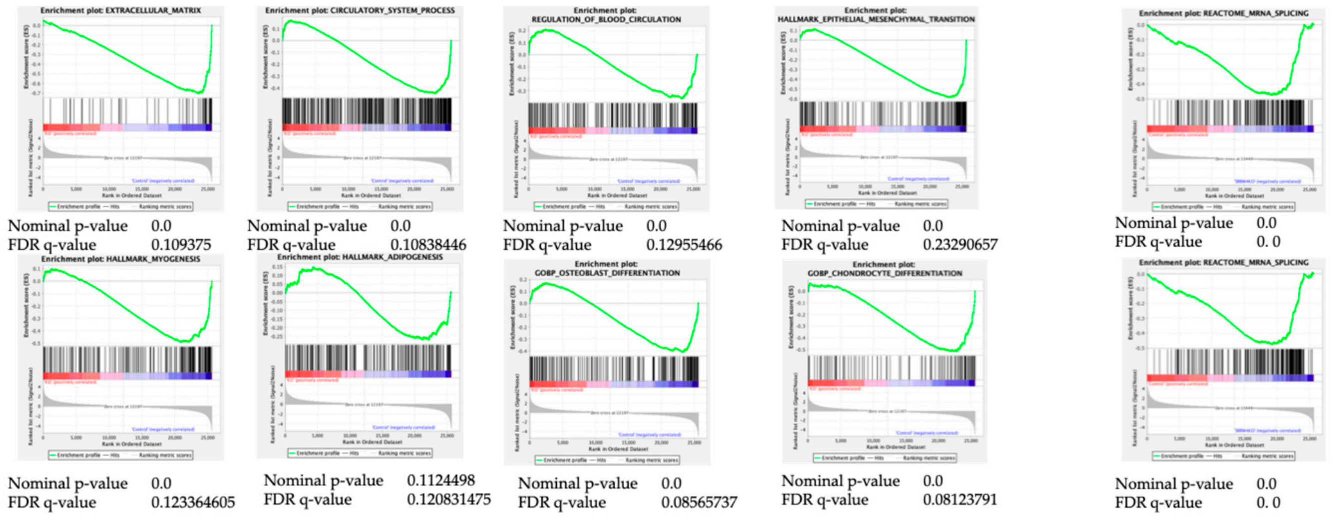

Figure S2. Graphical views of the enrichment plot of the GSEA

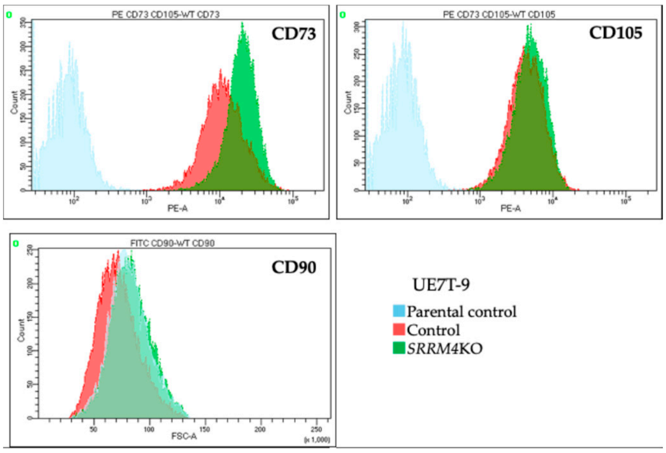

Figure S3. Validation of differentiation of UE7T-9 SRRM4 KO cell.

A. Flow cytometry analysis of CD73, CD105 and CD90.

Table S1. Antibodies used for flow cytometry analysis

target	fluorochrome	clone name	supplier	isotype	clonarity	host
SRRM4	none	bs-8069R	Bioss	IgG	polyclonal	Rabbit
CD73	PE	AD2	BD Pharmingen™	IgG1	monoclonal	Mouse
CD90	None	MRC OX-7	abcam	IgG1	monoclonal	Mouse
CD105	PE	SN6h	BD Pharmingen™	IgG1	monoclonal	Mouse
Mouse Ig	FITC	F0479	Agilent		polyclonal	Goat
Rabbit Ig	FITC	F0054	Agilent		polyclonal	Swine

Table S2. Geneset members on the rank-ordered list of GO enrichment analysis of differential genes

GOID	Description	GeneRatio	BgRatio	pvalue	padj	Count
GO:0008015	blood circulation	94/1566	411/14095	3.87E-12	1.55E-08	94
GO:0003013	circulatory system process	94/1566	414/14095	6.01E-12	1.55E-08	94
GO:0030198	extracellular matrix organization	74/1566	303/14095	3.13E-11	5.38E-08	74
GO:0042330	taxis	101/1566	479/14095	9.58E-11	1.24E-07	101
GO:0006935	chemotaxis	100/1566	478/14095	1.89E-10	1.95E-07	100
GO:0007059	chromosome segregation	77/1566	338/14095	4.34E-10	3.74E-07	77
GO:0140014	mitotic nuclear division	63/1566	256/14095	6.65E-10	4.90E-07	63
GO:0043062	extracellular structure organization	76/1566	341/14095	1.62E-09	1.05E-06	76
GO:0000280	nuclear division	80/1566	376/14095	5.83E-09	3.34E-06	80
GO:0098813	nuclear chromosome segregation	66/1566	290/14095	8.06E-09	4.16E-06	66
GO:0000819	sister chromatid segregation	56/1566	232/14095	1.24E-08	5.81E-06	56
GO:0030335	positive regulation of cell migration	83/1566	405/14095	1.85E-08	7.95E-06	83
GO:0048285	organelle fission	85/1566	420/14095	2.28E-08	9.04E-06	85
GO:0044057	regulation of system process	82/1566	402/14095	2.81E-08	1.02E-05	82
GO:0051272	positive regulation of cellular component movement	86/1566	429/14095	2.97E-08	1.02E-05	86
GO:0030900	forebrain development	67/1566	306/14095	3.16E-08	1.02E-05	67
GO:2000147	positive regulation of cell motility	84/1566	419/14095	4.30E-08	1.30E-05	84
GO:0050673	epithelial cell proliferation	68/1566	322/14095	1.10E-07	3.15E-05	68
GO:0040017	positive regulation of locomotion	86/1566	445/14095	1.64E-07	4.46E-05	86
GO:0000070	mitotic sister chromatid segregation	39/1566	148/14095	1.76E-07	4.53E-05	39
